# Alpine Summer Surface Temperature Amplification Is Spatially Heterogeneous and Intensified by Wind and Sun

**DOI:** 10.1002/ece3.72542

**Published:** 2025-11-23

**Authors:** Alexa A. C. MacDonald, Diana Stralberg, Scott E. Nielsen

**Affiliations:** ^1^ Department of Renewable Resources University of Alberta Edmonton Alberta Canada; ^2^ Northern Forestry Centre, Canadian Forest Service Natural Resources Canada Edmonton Alberta Canada

**Keywords:** alpine, microthermal heterogeneity, surface temperature amplification

## Abstract

Recent studies suggest that alpine microtopography may buffer against climate warming by creating more diverse climates over small spatial extents. Here, we examined with thermocouples how daytime summer surface temperatures differed from free‐air temperatures and how much summer diel temperatures varied across small areas (8 × 8 m) for eight paired (open and krummholz) alpine plots at Cardinal Divide, Alberta, Canada. We found summer daytime surface temperature amplifications with open plots averaging 26.7°C ± 0.2 SE on the ground relative to 21.1°C ± 0.1 SE at a 2‐m free‐air height (5.6°C ± 0.1 SE amplification). Krummholz plots decreased surface amplifications, compared to those observed in the open alpine, averaging 27.3°C ± 0.2 SE on the ground relative to 22.9°C ± 0.1 SE at 2 m (4.4°C ± 0.1 SE amplification). Wind and solar radiation altered temporal patterns in surface temperatures, resulting in increased amplification and decoupling from free‐air temperatures. At wind speeds of 3 m/s and solar radiation of 900 W/m^2^, surface temperatures in open alpine plots were up to 21°C higher than free‐air temperatures. Surface temperature variability over diel periods within 8 × 8 m plots differed by up to 9.9°C, illustrating high local variation in microclimates at a similar magnitude as the dry‐air adiabatic lapse rate for a 1‐km change in altitude. Individual locations with krummholz cover had diel ranges 11.3°C less than locations with no tree cover. Given the surface amplification and local spatial variability that we found, the use of standard 2‐m free‐air temperature data in climate models for alpine plants represents a mismatch in scales. More work is needed to develop climate surface models for alpine environments to assess risk from climate warming and determine opportunities for local refugia.

## Graphical abstract

Recent studies suggest that local topography drives spatial patterns in near‐surface microclimates of the alpine that scale with patterns in alpine plants. We compared summer daytime surface temperature amplifications (vs. free air temperatures) for small (8 × 8 m) open alpine and krummholz sites at Cardinal Divide, Alberta, Canada finding 5.6°C (± 0.1 SE) amplifications for surfaces at open sites and 4.4°C (± 0.1 SE) at krummholz sites. Mean diel surface temperatures within individual plots varied by up to 9.9°C, illustrating high local spatial variation and thus a need for surface climate models that can be used to better assess risks from climate warming and opportunities for local refugia in alpine ecosystems.

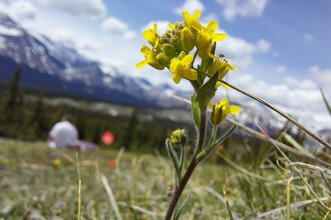

## 1 Introduction

Alpine ecosystems have highly variable microclimates, to which alpine plants are strongly coupled (Körner and Hiltbrunner 2021). These ecosystems are warming at a higher rate than ecosystems at lower altitudes and latitudes (Pepin et al. 2015). Future warming will shift microthermal habitats, thus altering species composition and diversity within mountain ecosystems (Scherrer et al. 2011; Körner and Hiltbrunner 2021). Free‐air temperature, commonly measured at a 2‐m weather station height (Scherrer et al. 2011), is often used in studies of alpine ecosystems, including projecting the effects of climate change on alpine plant species (Pauli et al. 2003). However, 2‐m free‐air temperatures may be disconnected from surface and shallow soil temperatures, which are known to most influence the growth and survival of alpine plants (Löffler and Pape 2020). More work is therefore needed to understand alpine surface temperatures and their relationship to alpine plants (Körner 2021).

Temperature is a key driving factor for species' fundamental niches (Löffler and Pape 2020). Billings (1973, 1974) considers three thermal scales within the alpine: macro‐gradients (latitudinal and altitudinal), meso‐gradients (dependent upon topography), and micro‐gradients (which can be created and influenced by something as small as an individual rock). Microclimates are formed via interactions between regional conditions and local topography (Dobrowski 2011). Alpine areas are typified by having high topographic variability, creating local microclimates associated with plants living close to the surface (Scherrer and Körner 2011). Greater topographic variability thereby creates greater variability in microclimates (Opedal et al. 2015), which creates more temperature niches, allowing for a more varied assemblage of plant species (Ohler et al. 2020). However, microclimates are not solely influenced by terrain. Wind and solar radiation also shape alpine microclimates, interacting with each other as well as with exposure and slope (Körner 2021). Wind influences the surface boundary layer and evaporative cooling effects, with alpine ecosystems typified as being windy environments. Finally, the plants themselves can shape the microclimates around them, influencing the degree of decoupling from free‐air temperatures (Körner 2021). Microclimatic variation in alpine environments has been shown to vary locally by up to 8°C, depending on the species assemblages and meso‐ to macroscale environmental conditions (Körner and Hiltbrunner 2021).

At the mesoscale, temperatures are often buffered under trees compared to an open reference condition (Rist et al. 2020). The encroachment of trees into the alpine, including krummholz patches of stunted trees, has been the focus of alpine climate change studies, since warming has been expanding the treeline (Körner and Paulsen 2004; Smith et al. 2009; Verrall and Pickering 2020; Körner 2021). Given expected upslope alpine treeline migrations (Kharuk et al. 2022) and the expansion of krummholz patches (Klasner and Fagre 2002; Dai et al. 2017), understanding how treeline transitional zones and krummholz influence surface thermal environments is key to understanding future alpine environments, including alpine plant responses.

Recent studies suggest that the microtopography and resulting microclimates present in the alpine zone may act as a buffer against climate warming (Scherrer and Körner 2010, 2011; Scherrer et al. 2011; Opedal et al. 2015; Ohler et al. 2020). Buffering of surface plants provided by microclimates, especially that due to tree cover, is often associated with a cooling effect in the daytime (Finocchiaro et al. 2023). However, in the alpine, surface temperatures during the day are often warmer than free‐air temperatures (Körner 2021), a phenomenon referred to as daytime amplification (Kotlarski et al. 2023). In contrast, the phenomenon of decoupling occurs when the rate of temperature change between the surface and free‐air differs (Lenoir et al. 2017). Plants at the surface are expected to be more decoupled from free‐air temperatures due to their small stature and proximity to the ground, leading to a lack of evaporative cooling (Körner 2021). These buffering and decoupling effects, as well as most alpine plants being stress‐tolerant (Grime 1977), suggest that alpine plants may be less susceptible to climate change than previously thought (Beniston and Haeberli 2001; Pauli et al. 2003), even if the alpine is warming faster than most other ecosystems (Scherrer and Körner 2010, 2011; Scherrer et al. 2011; Opedal et al. 2015; Ohler et al. 2020).

To anticipate how climate change may affect future alpine plant communities, we need to understand the microclimates they inhabit and the factors that influence them at the scale at which they occur. The goal of this study is to: (1) examine the extent of buffering, daytime amplification, and/or decoupling of surface alpine temperatures (vs. free‐air) with and without the presence of trees (krummholz), (2) determine how daily weather conditions alter these relationships, and (3) measure how spatially variable daily temperatures are at the surface, where most alpine biodiversity resides. Each objective has a series of predictions that we describe in more detail in Table 1.

**TABLE 1:** Study objectives and associated predictions regarding how daytime summer surface temperatures differ from free‐air temperatures (amplification vs. buffering) and how much summer diel temperatures vary across small scales (8 × 8 m) for eight paired (open and krummholz) alpine plots at Cardinal Divide, Alberta, Canada.

Objective	Predictions
1. Measure the scale of daytime surface temperature *amplification* during summer compared to free‐air temperatures for krummholz and open alpine plots	Amplification of daytime surface temperature (vs. free air) will be greater in open than krummholz alpine plots since trees will attenuate the scale of daytime surface warming due to shading of incoming solar radiation
2. Examine how *daily weather* conditions, including wind and solar radiation, can *increase amplification* rates of surface temperatures for open and krummholz alpine plots	Windy and sunny conditions will increase the amplification of surface temperatures, as the boundary layer effect at the surface will decrease the wind speed relative to free air, allowing for greater heating due to solar radiation. This effect should be attenuated in krummholz plots due to shading and obstruction of wind
3. Determine the *spatial variability* of diel surface temperatures across 8 × 8 m areas in paired open and krummholz alpine plots	Surface temperatures across an 8 × 8 m area will be highly variable, but less so in krummholz than in open plots. Surface temperatures in krummholz plots will also be more like free‐air temperatures than those in open plots, as trees will shade the surface and limit amplification

## 2 Methods

### 2.1 Study Site

We examined alpine microclimates at Cardinal Divide, near Cadomin in the Nikannassin Range of the Rocky Mountains in west‐central Alberta, Canada. Cardinal Divide is located within Whitehorse Wildland Provincial Park (WWP; 5763 ha), which straddles the Arctic (Athabasca River) and Hudson Bay (North Saskatchewan River) watersheds. The geology of WWP is sedimentary limestone, dolomite, sandstone, and shale (Achuff 1984), with the characteristic rugged landscape of the Canadian Rockies due to past glacial activity (Dinwoodie et al. 2020). Elevations range from 1840 m in valleys to 2755 m at the peak of Prospect Mountain, with the lowest elevations of the treeline at around 1950 m, but there are patches of tree cover on some slopes reaching 2150 m. The sedimentary substrate results in a more neutral soil pH, with many open alpine areas typified by exposed bedrock (Achuff 1984), restricting the rate and location of tree expansion. Historical records have documented 277 vascular plant species on the divide, which is unique in having a high local diversity of alpine plants and the presence of several disjunct arctic‐alpine species (Achuff 1984), suggesting a potential refugium in the last glacial period (Packer and Vitt 1974).

### 2.2 Study Design

**FIGURE 1:**
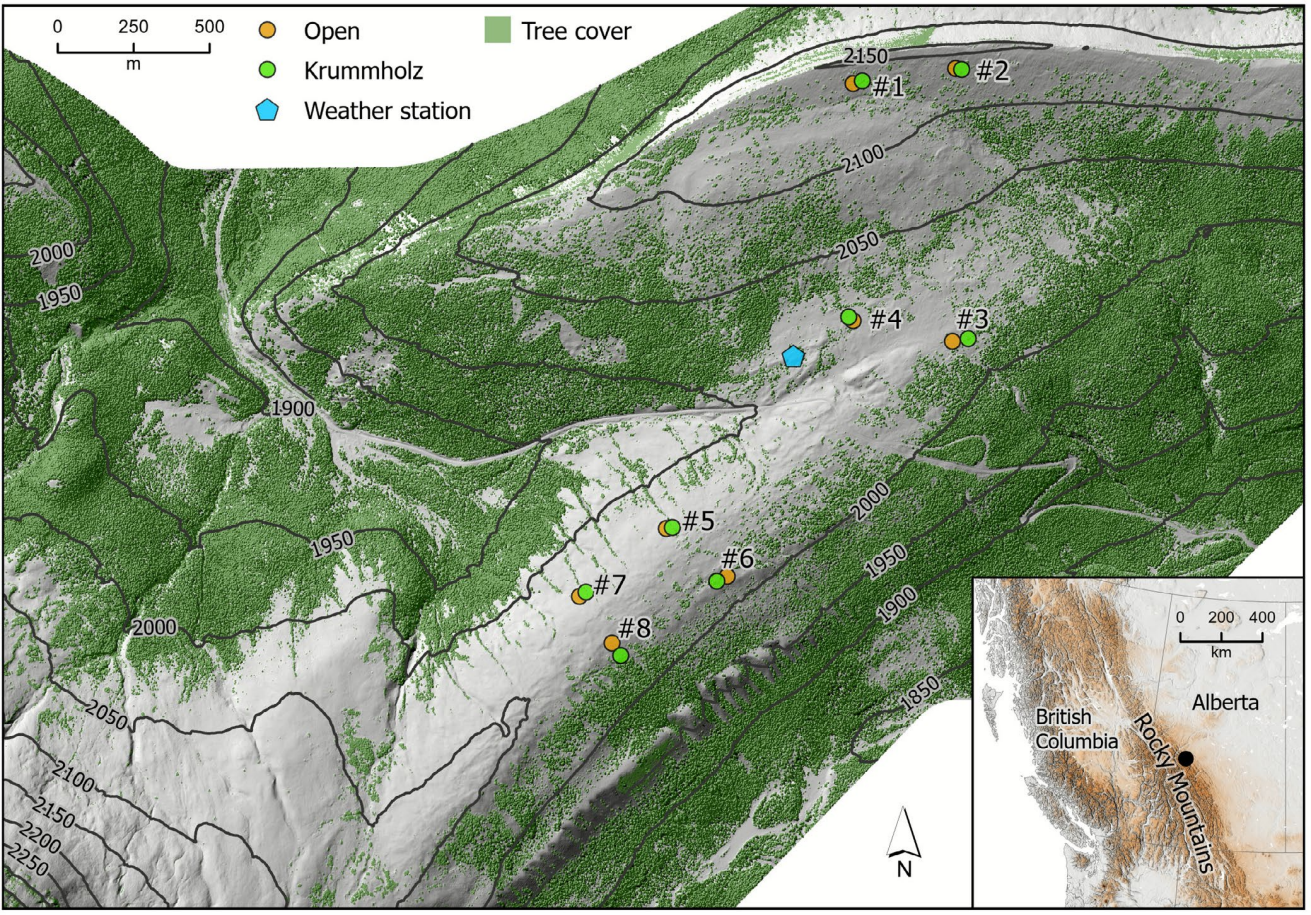
Map of our study area, Cardinal Divide, Alberta, Canada. Points on the map show the locations of the eight paired (open and krummholz) plots, and the centrally located weather station (respectively, gold circles, green circles, and blue pentagon). The base layer is a hillshade digital surface model with 50‐m increment contour lines in dark gray. The inset map illustrates the location of the Cardinal Divide (black point) within the Canadian Rocky Mountains (orange shading represents higher altitudes) and the terrain of western Canada and the adjacent northwest United States.

We established a series of paired plots in early June 2024 at eight sites across the divide, representing different parts of the terrain common to the area. This included flat areas, as well as more southern and northern exposures, all approximately at the same elevation (2000–2150 m) to control for altitude (Figure 1). Each pair consisted of one open and one adjacent krummholz plot within 30 m of each other and at the same elevation and slope to ensure consistency in other factors beyond the presence of trees. All plots were 8 × 8 m in size, placed with the edges facing cardinal directions (Figure 1). Plot size was selected to maximize the area covered while maintaining a sampling density adequate to provide data at the resolution required to meet the objectives of this study. Open plots were selected to have minimal shrub cover (prostrate shrubs may be present) and no direct canopy cover. Krummholz plots were selected as nearby isolated patches of trees or areas near the treeline. Each plot was initially surveyed using measuring tapes and a compass, and each sample location was marked with pin flags for temperature remeasurements during the remaining summer months (see Figure S1.1 for more details).

### 2.3 Field Methodology

#### 2.3.1 Vertical Temperature Measurements of Daily Surface Amplification in Summer

Each of the sixteen 8 × 8 m plots consisted of 145 within‐plot measurement locations spaced with specific patterns to capture the microthermal environments present in open and krummholz plots (see Figure S1.1 for a diagram of measurement locations). All temperature measurements at the 2320 locations (16 plots × 145 within‐plot locations) were recorded on sunny days from mid‐June to mid‐August of 2024 using an 8‐channel thermocouple (Huato S220‐T8) to allow for multiple vertical height measurements of temperature at the same time, recorded to a precision of 0.1°C. Thermocouples were fiberglass‐coated K‐type wires attached to a PVC pole using metal L‐brackets such that the end of the wire protruded beyond the end of the bracket so as not to interfere with the readings. Use of thermocouple wires to measure surface temperatures is recommended by Maclean et al. (2021) as they are small (< 1 mm), made of reflective material, and are thermally isolated from the recording unit. As recommended by Maclean et al. (2021), thermocouple wires were not shielded, although comparisons of shielded to non‐shielded thermocouple wires at a 2‐m height across eight sampling days showed that non‐shielded thermocouples averaged 1.1 (± 0.1°C) higher temperatures and were not sensitive to changes in ambient temperature (Table S2.1, Figure S2.1). Thermocouple wires were spaced at multiple heights to measure vertical temperature heterogeneity. However, here we focus on surface (0 cm) and free‐air (200 cm) temperatures to examine the surface amplification question specifically. The thermocouple unit recorded temperatures across continuous periods with the sample rate set to record every 2 s. Temperature samples were recorded for several seconds at each location to get a minimum of one, but in most cases several temperature recordings. For locations with multiple recordings, the last recording was used for analysis, assuming that the last thermocouple record would be most acclimatized to the locale's microclimate. A few sample locations within a plot (ten of the preselected 2320) had extremely dense krummholz cover, where measurements with the thermocouple pole were not possible. In these cases, corresponding locations in the paired open plot were removed before analysis to ensure equal sample sizes between open and krummholz paired plots.

#### 2.3.2 Reference Weather Station

We used a remote weather station established in 2022 at Cardinal Divide, located at an elevation of 2050 m and in the middle of our research plots, to measure free‐air temperature, wind speed, and solar radiation at a 2 m height. The station is situated on a flat site with no direct tree cover (see Figure 1 for location). Free‐air temperature was measured using a Hobo temperature sensor (E348‐S‐THB‐M002), with a solar radiation shield (E348‐RS3‐B). Wind speed was measured using a Davis Wind Speed and Direction Smart Sensor (E348‐S‐WCF‐M003). Solar radiation was measured using a silicon pyranometer sensor (E348‐S‐LIB‐M003). The sampling frequency was 15 min. We used weather station data for objectives two and three. Data used for assessing surface amplification from weather variables in objective two of the study were based on the closest matched times of point measurements (0‐m and 2‐m temperature measurements). Ambient air temperature data from the weather station were also used as a comparison in the horizontal surface microthermal heterogeneity analysis for objective three, where measurements were recorded and used across the same 24‐h periods.

#### 2.3.3 Microthermal Horizontal Heterogeneity

Thermocouple sensors were deployed simultaneously in each of the paired plots once during the summer for a minimum of one diel (24‐h) period that was representative of typical summer weather conditions (< 1.5 mm of rain). Each of the paired plots had six four‐channel thermocouple (Perfect Prime TC0520) units, allowing for 24 fiberglass‐coated K‐type wires to be systematically spaced throughout each plot (Figure S1.1) and used to record diel surface temperatures at 30‐s intervals. This resulted in 69,120 samples per location and 24 locations per plot. Thermocouple wires were unshielded as per recommendations by Maclean et al. (2021) and were placed within 1 cm of the surface. At locations with dwarf shrub growth, wires were placed within or under the shrub, as close to the surface as possible. We used 50 cm × 50 cm quadrats to measure alpine plant cover, and the cover of exposed rock and bare soil around all 24 sensor locations per plot.

### 2.4 Data Analysis

#### 2.4.1 Surface Temperature Amplification

**FIGURE 2:**
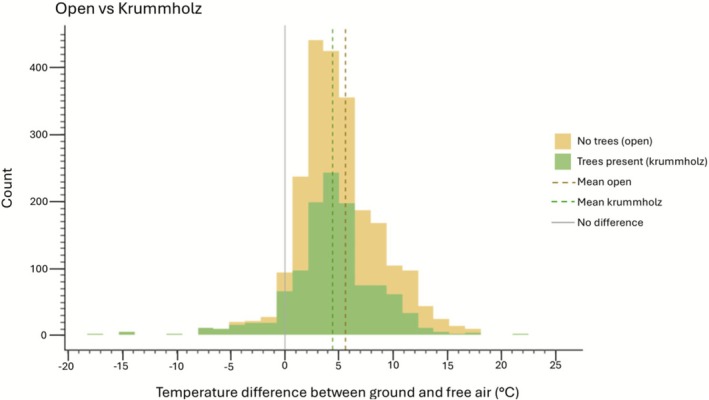
Histograms illustrating the difference in temperatures between surface and free air across all 16 plots, split according to the presence of krummholz trees (*n* = 8) and open alpine (*n* = 8). Measurements were taken in paired plots on sunny days from mid‐June to mid‐August. Figure displays both within‐plot and between‐plot variation (for graphs without between‐plot variation, see Appendices S5 and S6). The mean of each histogram (krummholz or open) is shown as a dashed line. Surface and free air at equilibrium (0°C) is shown as a solid gray line; the area left of this represents surface cooling; the area to the right represents surface warming.

We used a one‐sample *t*‐test to investigate the daytime amplification effect of surface temperatures (vs. 2‐m free‐air) at krummholz and open plots. Here, a difference between surface and free‐air temperatures of 0°C indicates no amplification or buffering (surface temperatures match 2‐m free‐air temperatures). Values > 0°C represent surface temperature amplification, while values < 0°C represent surface buffering. We combined all pairs together to examine the overall effect of tree cover (Figure 2; Table S3.1). We then used a Welch's two‐sample *t*‐test for unequal variances to determine if amplification differed between open and krummholz plots (Figure 2; Table S3.1). Additionally, one‐sample *t*‐tests were conducted separately for each pair (open vs. krummholz plots) using the same trial date to examine the influence of plot type on the amplification effect (Figure S4.1).

#### 2.4.2 Weather‐Related Amplification of Surface Temperatures

We further assessed the effects of daily weather on the difference between surface and 2‐m free‐air temperature in open and krummholz plots using generalized linear mixed effect models (GLMM) and purposeful model selection (Bursac et al. 2008) based on a series of free‐air (2‐m height) weather variables. Weather variables included solar radiation (initial measurements were divided by 100, resulting in units of W/100 m^2^), wind speed (m/s), and free‐air temperature (°C), and any significant interactions (*p* < 0.05). We fit both the open and krummholz models with raw and normalized units (Ali and Faraj 2014), with normalized variables ranging from 0 to 1. The response variable was the difference between surface temperature and 2‐m free‐air temperature measured at each location (Gaussian family), representing the surface amplification or buffering effect. Plot pair was used as a random effect to account for correlations within plots.

#### 2.4.3 Microthermal Horizontal Heterogeneity

For each surface temperature trial within plots, we selected the first whole 24‐h (diel) period (12:00:00 AM to 11:59:59 PM). For each of the 24 locations per plot, we calculated the mean 24‐h surface temperature and temperature range over that period (Figure 1). We then calculated the overall mean and 95% confidence intervals for the daily mean temperature and range for each of the 16 plots (8 open‐to‐krummholz pairs) to estimate the average daily summer surface climate for each 8 × 8 m plot. To assess significant differences, we simply examined whether the confidence intervals of the diel range of temperatures between pairs of open and krummholz plots overlapped with one another, as well as with the nearby weather station's free‐air temperatures for that sampled diel period. Each pair was kept separate since each pair had a different trial date, using temperatures in comparisons that were unique to that period.

We further assessed the relationship between temperature maximum, minimum, and range values and surface cover using a GLMM and purposeful model selection (Bursac et al. 2008) based on broad ground cover categories. These categories included the percent cover of bare soil, bare rock, graminoids (e.g., grass species, *Juncus* spp., and *Carex* spp.), shrubs (excluding dwarf shrub species of *Salix nivalis* , *Juniperus communis* , *Salix arctica* , *Arctous rubra* , *Arctostaphylos uva‐ursi* , *Cassiope tetragona* , and *Phyllodoce glanduliflora* ), trees, herbaceous and prostate plants (including the above‐listed species), and cryptograms (e.g., bryophytes, lichens, and cryptobiotic crust). Random effects were the date of trial and the presence or absence of trees (krummholz vs. open binary variable).

## 3 Results

### 3.1 Surface Temperature Amplification

Mean surface temperature differences from that of free‐air were significantly > 0°C for both open (5.6°C ± 0.1 SE) and krummholz (4.4°C ± 0.1 SE) plots, indicating a daytime alpine surface amplification (Figure 2; Table S3.1). Although surface temperatures of both open and krummholz plots were amplified and widely overlapped, the presence of trees decreased the amplification by an average of 1.2°C (*p* < 0.001; 95% CI [0.9, 1.6]—see Table S3.1), demonstrating a weak, but noticeable reduction in surface temperature amplification at the scale of the plot.

### 3.2 Weather‐Related Amplification of Surface Temperatures

**FIGURE 3:**
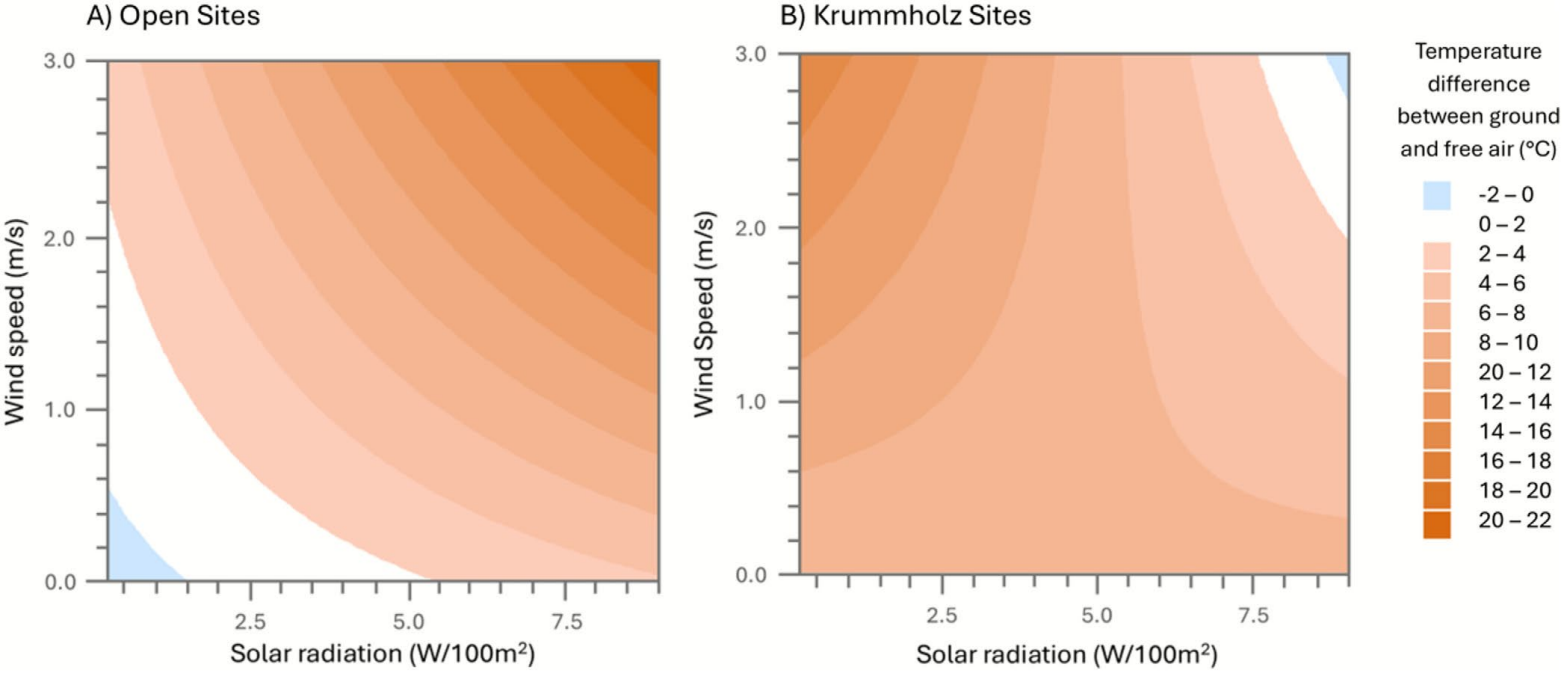
Predicted alpine summer daytime surface temperature amplification to free‐air (2‐m) temperatures at Cardinal Divide, Alberta, Canada, given wind speed (m/s) and solar radiation (W/100 m^2^), holding ambient air temperature at its mean (14.8°C for open alpine and 16.3°C for krummholz treed). (A) shows open alpine plots, while (B) shows alpine krummholz plots. Orange shading indicates a surface amplification effect, with darker shades indicating greater strength of warming. White indicates minimal amplification (0°C–2°C). And finally, blue shading indicates a surface cooling effect, with darker shades indicating greater strength of cooling.

The combined (interactive) effects of solar radiation and wind speed resulted in the largest changes in surface temperature amplification of open alpine plots (β = 0.52; *p* = 0.002; Table 2). Days with higher wind speeds and solar radiation had even greater amplification of daytime surface temperatures (Figure 3A). For instance, at wind speeds of 3 m/s and solar radiation of 9 W/100 m^2^, we found up to a 21°C difference between surface and free‐air temperatures in open plots (Figure 3A). In krummholz plots, the direction of the effect was opposite (β = −0.64; *p* < 0.001; Table 2) (Figure 3B), and predicted effects were more variable. For example, at solar radiation levels of 0.2 W/100 m^2^ and wind speeds of 2.7 m/s, we found warming of up to 16°C in krummholz plots; however, as solar radiation levels increased to 9 W/100 m^2^, holding wind at 3 m/s, the amplification effect of surface temperatures was lost (Figure 3; Table 2).

**TABLE 2:** GLMM (Gaussian family) results illustrating the influence of daily weather on surface temperature amplifications in (A) open and (B) krummholz alpine plots at Cardinal Divide, Alberta, Canada.

Variable	Open alpine					Krummholz alpine				
	β	SE	β_norm_	*Z*	*p*	β	SE	β_norm_	*Z*	*p*
Intercept	−9.51	2.49	−7.46	−3.82	< 0.001	−14.58	4.37	−10.28	−3.34	< 0.001
Free‐air temperature	0.59	0.11	12.87	5.49	< 0.001	1.26	0.36	27.36	3.54	< 0.001
Solar radiation	1.21	0.30	9.36	4.05	< 0.001	2.29	0.65	16.10	3.54	< 0.001
Wind speed	1.07	0.73	3.60	1.47	0.141	−2.92	2.14	−5.38	−1.37	0.172
Solar radiation × wind speed	0.52	0.17	13.82	3.11	0.002	−0.64	0.19	−16.99	−3.31	< 0.001
Solar radiation × free‐air temperature	−0.05	0.02	−9.34	−2.14	0.032					
Wind speed × free‐air temperature						0.42	0.13	28.05	3.20	0.001
Free‐air temperature × solar radiation						−0.15	0.06	−29.28	−2.49	0.013

*Note:* Weather variables included free‐air (2 m) temperature (°C), solar radiation (W/100 m^2^), and wind speed (m/s). Plot pair was used as a random effect to account for correlations within plots.

### 3.3 Surface Horizontal Microthermal Heterogeneity

**FIGURE 4:**
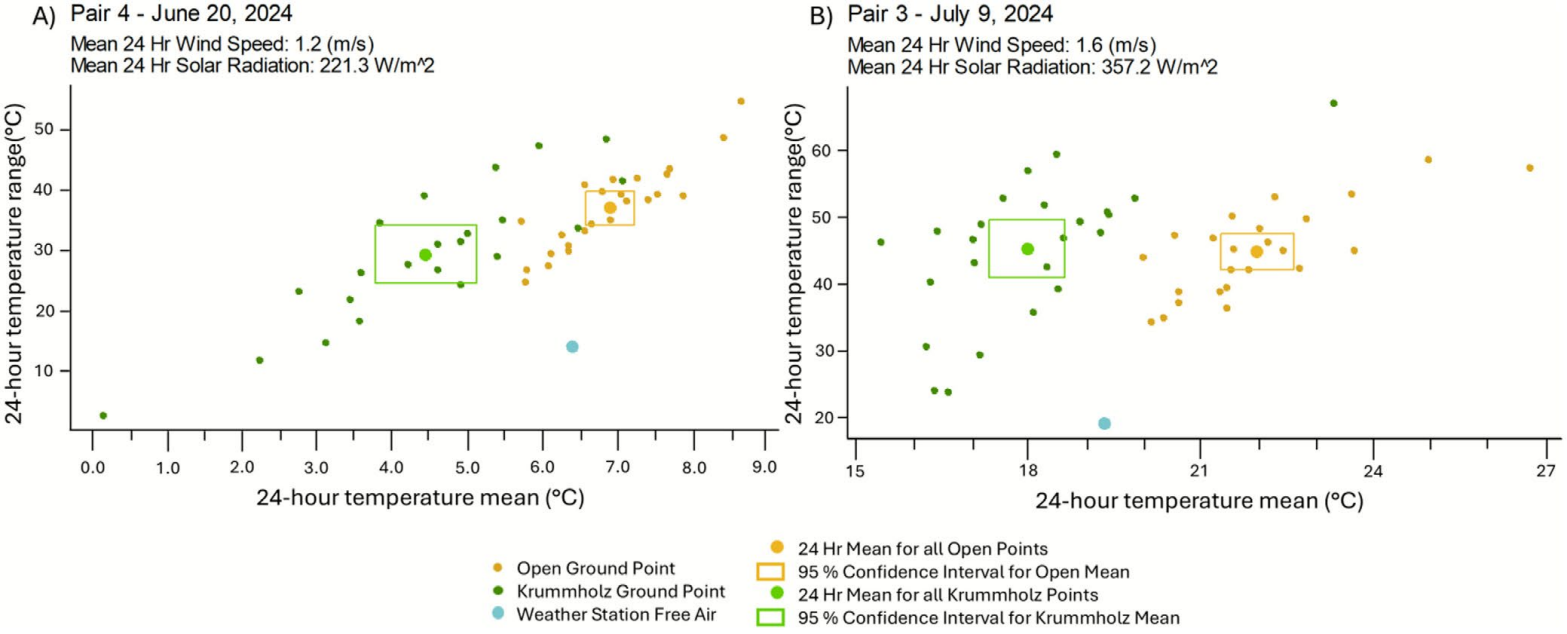
Spatial variation in the diel (24‐h) range and mean surface temperatures for paired open and krummholz plots (8 × 8 m) with 24 sample locations each. In (A), we demonstrate the conditions for a pair of plots from a cool summer day (mean free‐air temperatures of 6.4°C), while in (B), we demonstrate the conditions for a pair of plots from a hot summer day (mean free‐air temperature of 19.3°C). The points are color‐coded such that gold represents plots without trees (open) and green represents plots where trees are present (krummholz). The overall mean and range of temperatures for open and krummholz locations are shown as larger points of the same color, with the 95% confidence intervals represented as a bounded box around those points. The mean and range of free‐air temperatures for the same period were used as a standard reference and measured by a nearby weather station, indicated in the graphs as a single blue point.

Figure 4 shows the pairs of the open and krummholz plots measured on the days with the (A) lowest and (B) highest 24‐h (diel) mean free‐air temperatures (see Figures S5.1 and S5.2 for temporal curves of Pairs 3 and 4, respectively). Comparisons of raw thermocouple temperature measurements to those adjusted for a 1.1°C warming of the thermocouple wires (Table S2.1, Figure S2.1) found no change in the overall interpretation, so we report here the unadjusted values. Mean diel temperatures of the open and krummholz plots differed from the free‐air temperature of the nearby weather station (i.e., the 95% confidence intervals do not overlap with the free‐air temperature point), for all pairs except pair one (Figure S6.1). In all pairs except pair eight, mean surface temperatures of open alpine plots were significantly greater than the mean of the corresponding adjacent krummholz plot. For pair eight, the 95% confidence intervals overlapped slightly (Figure S6.1). As would be expected, the mean surface temperature range was often greater in open than krummholz plots, indicating greater temporal variability in temperatures across the 24‐h measurement periods (Figure 4; Figure S6.1). For example, in pair one, the mean diel temperature range for open plots was 31.9°C while the mean range for krummholz plots was 18.0°C (Figure S6.1). The largest diel surface temperature range observed was 73.7°C (minimum temperature of 2.4°C and maximum temperature of 76.1°C in plot eight‐open).

However, when considering the variability of individual locations within the same plot, krummholz plots showed greater variability. Across measurement locations in the same plot, the mean 24‐h surface temperature was found to differ by up to 7.3°C at locations in open plots (pair two average maximum of 16.4°C and average minimum of 9.1°C) and by up to 9.9°C at locations in krummholz plots (pair two average maximum of 15.7°C and average minimum of 5.8°C) (Figure S6.1). The difference in diel surface temperature ranges across locations in the open plot of pair four was 30.1°C (minimum of 24.8°C diel range measured at one location to maximum of 54.9°C measured at another location in the same 8 × 8 m plot); however, in the krummholz plot of pair four the difference in diel surface temperature ranges across locations was 45.9°C (minimum of 2.7°C diel range measured at one location to maximum of 48.6°C measured at another location in the same 8 × 8 m plot). This illustrates how variable the daily surface temperatures of the alpine can be both temporally (across a 24‐h period) and spatially (8 × 8 m).

Only tree and shrub ground cover significantly affected the maximum and range of diel temperatures at locations within plots. Here, there were strong buffering effects of trees with a 100% increase in tree cover predicted to reduce maximum temperatures by 9.9°C and increase minimum temperatures by 1.6°C, thus reducing the diel temperature range by 11.3°C (Table 3). The amount of rock cover most affected minimum diel temperatures, where a 100% cover led to a 2.2°C increase in minimum temperatures (Table 3).

**TABLE 3:** GLMM (Gaussian family) results illustrating the influence of the percent cover of vegetation types on (A) maximum surface temperature and (B) surface temperature range across a 24‐h (diel) period, measured at Cardinal Divide, Alberta, Canada.

Variable	Maximum temperature (°C)				Diel temperature range (°C)				Minimum temperature (°C)			
	β	SE	*Z*	*p*	β	SE	*Z*	*p*	β	SE	*Z*	*p*
Intercept	38.0	2.7	13.91	< 0.001	36.1	2.5	14.55	< 0.001	1.9	1.1	1.67	0.09
Trees	−9.9	1.0	−10.30	< 0.001	−11.3	1.4	−8.05	< 0.001	1.6	0.2	9.52	< 0.001
Shrubs	4.2	2.1	2.03	0.042	4.4	2.2	1.99	0.047				
Graminoids									−1.2	0.6	−2.11	0.035
Herbaceous plants + dwarf shrubs									−0.3	0.2	−2.25	0.025
Bare rock									2.2	0.4	5.28	< 0.001

*Note:* Vegetation cover types included tree species, shrub species (excluding the dwarf shrubs), graminoid species (e.g., grass species, *Juncus* spp., and *Carex* spp.), combined herbaceous plant species and dwarf shrubs, and bare rock. Random effects were the date of trial and the presence or absence of trees (krummholz vs. open binary variable).

## 4 Discussion

This study examined the differences between alpine surface (0 m) and free‐air (2 m) temperatures in summer, including the effects of krummholz patches of trees and daily weather, and how surface temperatures vary horizontally across small scales over diel periods. Although previous studies have examined alpine surface temperatures (Scherrer and Körner 2010; Gubler et al. 2011; Opedal et al. 2015), the magnitude and drivers of differences between surface and free‐air temperatures are not well known. We used a natural experimental comparison of open alpine and krummholz paired 8 × 8 m plots within 30 m of each other at the same elevation and slope to measure local temperature variability of alpine surfaces. Overall, we found surface amplification effects of 5.6°C in open patches and 4.4°C in krummholz patches. The amplification effect was strengthened in open areas under high wind and solar radiation levels. Surface temperatures were spatially heterogeneous across small 8 × 8 m areas, with mean diel surface temperatures varying by up to 9.9°C, a magnitude approximately equivalent to the dry air adiabatic lapse rate for a 1‐km change in altitude. Surface temperature amplification at krummholz plots was reduced (1.2°C) but varied substantially at the within‐plot scale (11.3°C). These results illustrate the high spatial variability of alpine surface temperatures at the scale at which alpine plants occur.

### 4.1 Surface Temperature Amplification

As is known from previous studies (Körner 2021), and shown here (Figure 2), daytime alpine surface temperatures in the summer are amplified when compared to free‐air temperatures. We demonstrated that summer surface temperatures in open plots were, on average, 5.6°C warmer (amplified) than free‐air temperatures. This amplification decreased to 4.4°C for krummholz plots, though a 1.2°C difference may not be biologically significant given that alpine plants here were experiencing daily temperature ranges of up to 73.7°C (minimum temperature of 2.4°C and maximum temperature of 76.1°C in plot eight open) (Figure S6.1). Thus, counter to our prediction, alpine krummholz, when examined at the plot level, did not substantially attenuate the amplification of alpine surface temperatures during summer. This contrasts with the influence of tree canopy in other environments. In a global analysis, De Frenne et al. (2019) found that forest cover buffers ground temperatures (and thus understory plants) under the canopy from those of free‐air temperatures in openings. The difference with our study may be due to the small size and short canopy heights of alpine krummholz patches, which can have gaps in the canopy where interior forest‐like conditions are not present. As such, at a plot scale, a significant buffering effect of krummholz was not present. However, nearby locations with greater canopy cover and tree height (e.g., subalpine forests) may result in cooler (buffered) conditions.

### 4.2 Weather‐Related Amplification of Surface Temperatures

We found a significant and interactive effect of wind and solar radiation on amplification of surface temperatures (Table 2; Figure 3). This was often substantial, with up to 21°C surface amplification at 3 m/s wind speed and solar radiation levels of 9 W/100 m^2^ in open alpine plots. Here, the wind was removing heat via convection at a 2‐m height, while the surface remained heated by high solar radiation levels. Surface temperatures can be shielded from winds due to the boundary layer effect (Bailey et al. 1997) and due to the presence of rocks and meso‐to‐micro terrain features altering surface winds and absorbing/radiating heat (Körner 2021). Temperate and arctic alpine environments are typified by high winds (Körner 2021), resulting in amplification effects like these being common.

The variability of tree density and canopy cover in krummholz plots creates unique microthermal environments that lack generalizations. For example, at solar radiation levels of 0.2 W/100 m^2^ and wind speeds of 2.7 m/s, we found warming of up to 16°C; however, as solar radiation levels increased up to 9 W/100 m^2^ and winds increased to 3 m/s, the amplification effect disappeared (~0°C, Figure 3; Table 2). Understanding the complexity of these alpine environments is important, as krummholz patches and subalpine treelines are expanding (Klasner and Fagre 2002; Dai et al. 2017).

The changes in amplification that we found between krummholz and open plots illustrate that weather measured at a standard meteorological station height influences surface and free‐air temperatures in different ways. In situ measurements of wind speed and solar radiation are needed to fully understand and predict their effects on surface temperatures. As such, care should be taken in using standard weather data and climate models for assessing the impacts of climate change on alpine plants.

### 4.3 Surface Horizontal Microthermal Heterogeneity

Beyond the vertical differences in free‐air temperatures, we found extreme horizontal microthermal heterogeneity at small scales (8 × 8 m). Krummholz plots typically had greater variability in surface temperatures (diel mean and range) than open plots. The 24‐h mean surface temperature differed in individual plots by up to 7.3°C in open plots and by up to 9.9°C in krummholz plots (Figure S6.1). This 9.9°C difference across a small 8 × 8 m plot is equivalent to an increase of 1.5 km in altitude using an average adiabatic lapse rate of 6.5°C/km, or 1 km in altitude using a dry air adiabatic lapse rate of 9.8°C/km (Barry and Chorley 2009). These results thereby demonstrate the extreme local microclimates experienced by alpine plants within a small area (Scherrer and Körner 2010). The greater variation in krummholz plots is counter to our prediction of buffering, which assumed more uniform temperatures in krummholz plots due to canopy shading and microclimate homogenization. The greater variability across locations within krummholz plots is likely due to the variability of canopy cover associated with krummholz, where locations in openings are shielded from wind yet exposed to solar radiation and surface warming, while locations under high canopy cover experience significant buffering of diel temperature ranges (Table 3).

We found that trees and shrubs cooled daytime maximum temperatures and thus reduced diel temperature ranges. Conversely, the effect of tree cover on minimum temperature was positive, aligning with other findings that forests buffer against extreme cold temperatures (Ferrez et al. 2011). When considering krummholz at the plot level, there was little buffering of surface temperature amplification. However, when under areas of high tree cover, amplification of surface temperatures was clearly reduced (diel range decreases by 11.3°C). This within‐plot variability further emphasizes that krummholz plots are not easily generalizable due to the heterogeneous conditions present.

The largest diel surface temperature range observed was 73.7°C (minimum temperature of 2.4°C and maximum temperature of 76.1°C in plot eight open). The free‐air temperature range for this date, as measured by the weather station, was 17.2°C. This location had cover values of 2% bare rock, 2% bare soil, 23% herbaceous plants and dwarf shrubs, and 48% moss, lichen, and cryptobiotic crust. This extreme temperature range may be due to a lack of shading (no shrubs, trees, or herbaceous plants) and a larger boundary layer, due to moss and lichen cover. Surface boundary layers protect the surface from winds while allowing the surface to heat under high amounts of solar radiation (Bailey et al. 1997).

## 5 Conclusion

An understanding of alpine microthermal environments requires in situ measurements of microclimatic conditions of the alpine surface, which can vary drastically within and across alpine habitats (Scherrer and Körner 2011). Our study documented daytime amplification of surface temperatures in the alpine (~5°C) and showed that krummholz patches, when considered as a whole, had minor effects on reducing temperature amplification (~4°C). When examining tree cover directly, locations with high tree cover experienced significant buffering of diel temperature ranges (~11°C), illustrating the complexity of microclimates in krummholz patches. Surface temperatures were highly variable across small (8 × 8 m) scales. Such horizontal variability increases the availability of microrefugia across the alpine (Löffler and Pape 2020). Given the strong surface amplification (which is increased by the effects of wind and solar radiation), the complexity of krummholz microclimates, and the horizontal variability present across small scales, the use of standard free‐air temperatures from climate models (i.e., macroclimate) to represent the environments experienced by low‐growing, small alpine plants is not likely to be representative and thus effective for understanding their ecology, thermal niches, and climate change risks. More work is needed to develop a better understanding of surface climates for alpine environments at the scale at which alpine plants exist.

## Author Contributions

**Alexa A. C. MacDonald:** conceptualization (supporting), data curation (lead), formal analysis (lead), investigation (lead), methodology (equal), project administration (equal), visualization (equal), writing – original draft (lead). **Diana Stralberg:** investigation (supporting), writing – review and editing (supporting). **Scott E. Nielsen:** conceptualization (lead), formal analysis (supporting), funding acquisition (lead), methodology (equal), project administration (equal), resources (lead), supervision (lead), visualization (equal), writing – review and editing (lead).

## Funding

This work was supported by NSERC grant RGPIN‐2019‐06040 Nielsen.

## Conflicts of Interest

The authors declare no conflicts of interest.

## Supporting information


**Appendices S1–S6:** ece372542‐sup‐0001‐AppendixS1‐S6.zip.

## Data Availability

The data that support the findings of this study are openly available in Cern's Data Centre (Zenodo) at https://zenodo.org/records/17309071, DOI: 10.5281/zenodo.17309071.
