# Supplementary material for: Alpine Summer Surface Temperature Amplification Is Spatially Heterogeneous and Intensified by Wind and Sun
Source: Ecol Evol. 2025 Nov 23;15(11):e72542. doi: 10.1002/ece3.72542 (PMC12640703; doi:10.1002/ece3.72542)
Supplement: Supplementary file 1 — Appendices S1–S6: ece372542‐sup‐0001‐AppendixS1‐S6.zip. [file ECE3-15-e72542-s001.zip › Appendix.docx]

**Appendix S1.** Layout of measurement points in each plot.


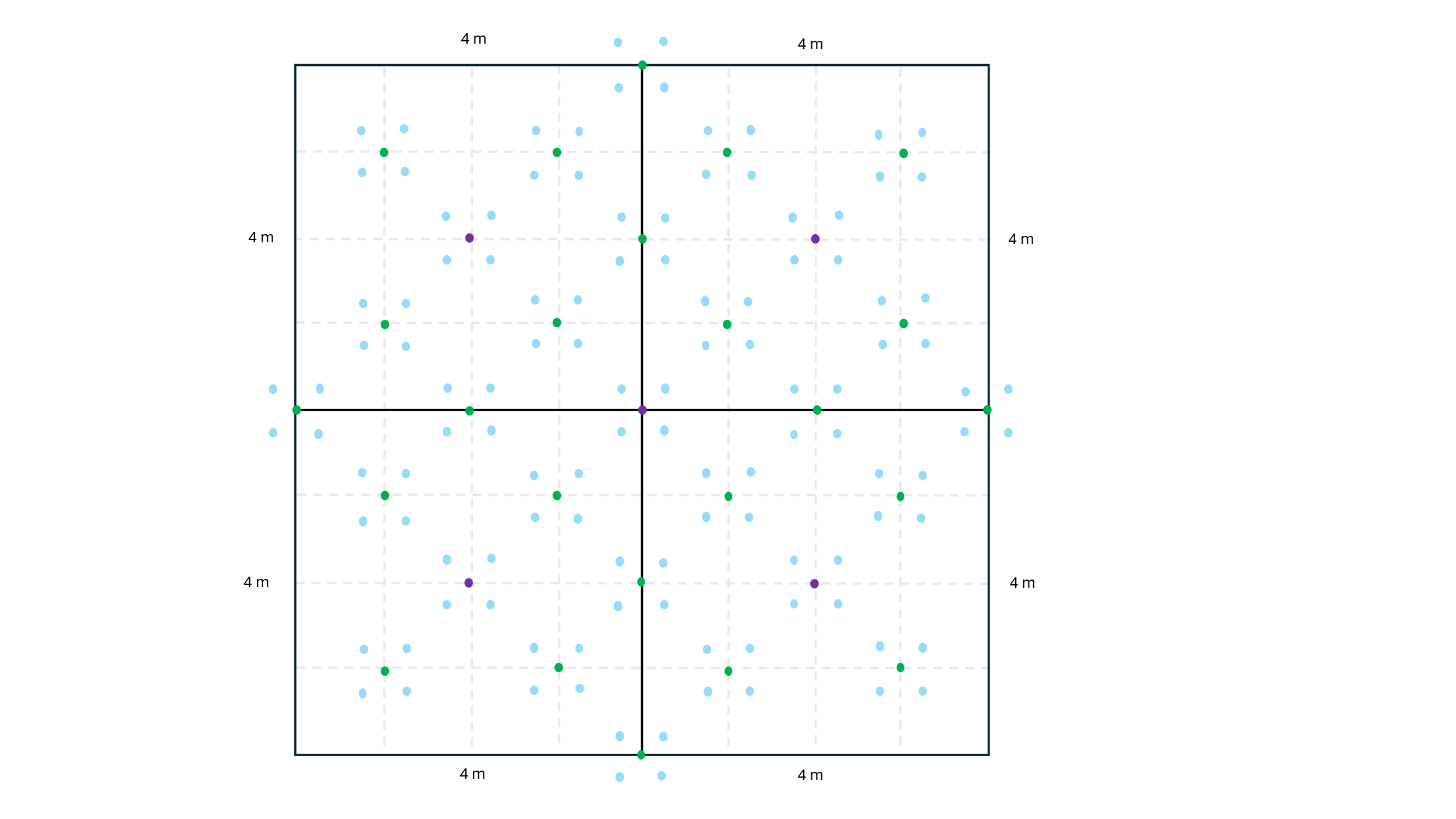


**Figure S1.1.** A diagram of measurement locations within each 8×8 m plot. The locations represented by purple and green points were pin-flagged. Locations represented by blue points were measured only once at 0 and 200 cm heights and found using a guide placed around each marked location. Thermocouples were placed on the alpine surface (< 1 cm) at locations represented by green points for a minimum of one diel period (24 continuous hours) for each pair (open and krummholz).

**Appendix S2.** Comparisons of open and shielded thermocouple measurements of free-air temperature.

Maclean et al. (2021) paper “On the measurement of microclimate” provides an overview of challenges of microclimatic measures and recommendations. Use of shields on the surface reduces wind speeds near the surface, thus limiting airflow and altering the temperature of the surface measured. Some types of temperature sensors (data loggers) may bias the temperature readings, especially if large and encased in plastic (e.g., HOBO loggers in PVC pipes, iButtons enclosed in plastic cases). Maclean et al. (2021) recommend the use of wire thermocouples to measure surface temperatures, noting that they reduce measurement errors the most. We followed these recommendations by using K-type thermocouple wires that were unshielded at all measurement heights to maintain consistency. This fits the recommended attributes: they are small (<1 mm), have a highly reflective surface (metal), and are thermally isolated from the recording unit.

Regardless, we took measurements of our thermocouple wires at the weather station to examine potential biases of the sensors by comparing unshielded and shielded thermocouple wires at a standard 2 m height. This was completed during the same 8 sample days used for measuring surface climates of the paired plots. Overall, we did observe a 1.1 °C (± 0.1) difference with unshielded sensors being warmer, consistent with Maclean et al. (2021). However, these differences were not related to ambient air temperatures (Figure B1), suggesting no systematic bias with changes in ambient temperatures.

**Table S2.1.** Results of the two-sample t-test comparing shielded and unshielded thermocouple wires at 2 m. The test site was the weather station at Cardinal Divide, Alberta, Canada. Test dates match the measurement dates for vertical point temperature measurements.

| **T-Test** | **Mean Shielded** | **Mean Unshielded** | **95% CI** | **DF** | **t** | **p** |
| --- | --- | --- | --- | --- | --- | --- |
| Shielded vs Unshielded | 18.3 | 17.2 | 0.2 – 1.9 | 573.88 | 2.49 | 0.013 |

**
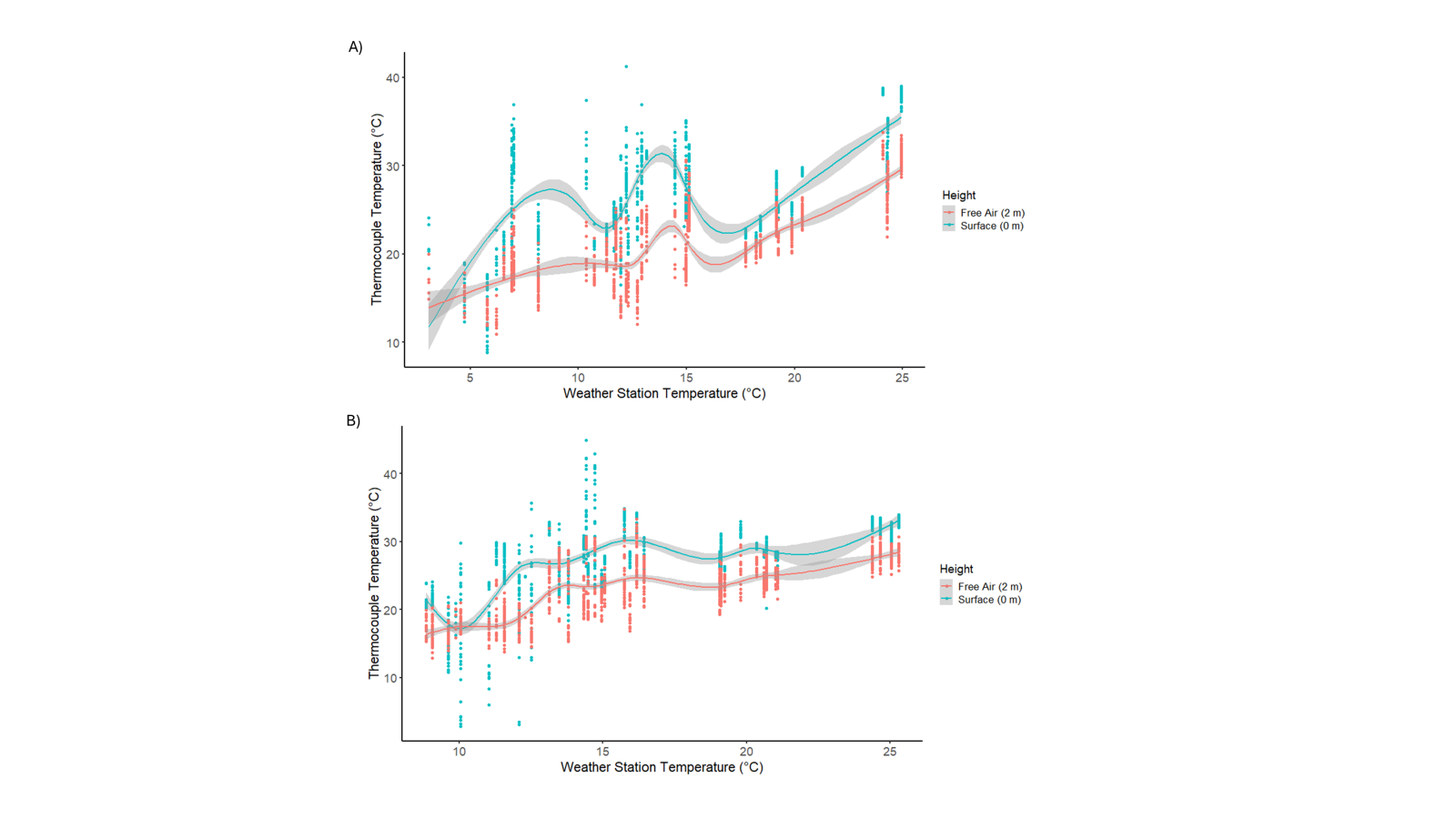
**

**Figure S2.1.** Plots showing the response of surface and 2 m temperatures to free-air (2 m) temperature recorded by the weather station in **A)** open plots and **B)** krummholz plots. Data were collected at Cardinal Divide, Alberta, Canada, from June to August 2024, on clear, sunny days.

**Appendix S3.** Results of t-tests for comparing open and krummholz surface temperature amplifications.

**Table S3.1.** Results of t-tests for open and treed plots.

| **T-Test** | **Mean X** | **Mean Y** | **95% CI** | **Degrees of Freedom** | **t-value** | **p-value** |
| --- | --- | --- | --- | --- | --- | --- |
| Open vs Krummholz | 5.6 | 4.4 | 0.9 – 1.6 | 2271.1 | 7.4 | < 0.001 |
| Open vs 0°C | 5.6 | N/A | 5.4 – Inf | 1149 | 50.1 | < 0.001 |
| Krummholz vs 0°C | 4.4 | N/A | 4.2 – Inf | 1149 | 35.0 | < 0.001 |

**Appendix S4.** Results of t-tests comparing open vs. krummholz paired plots.

**
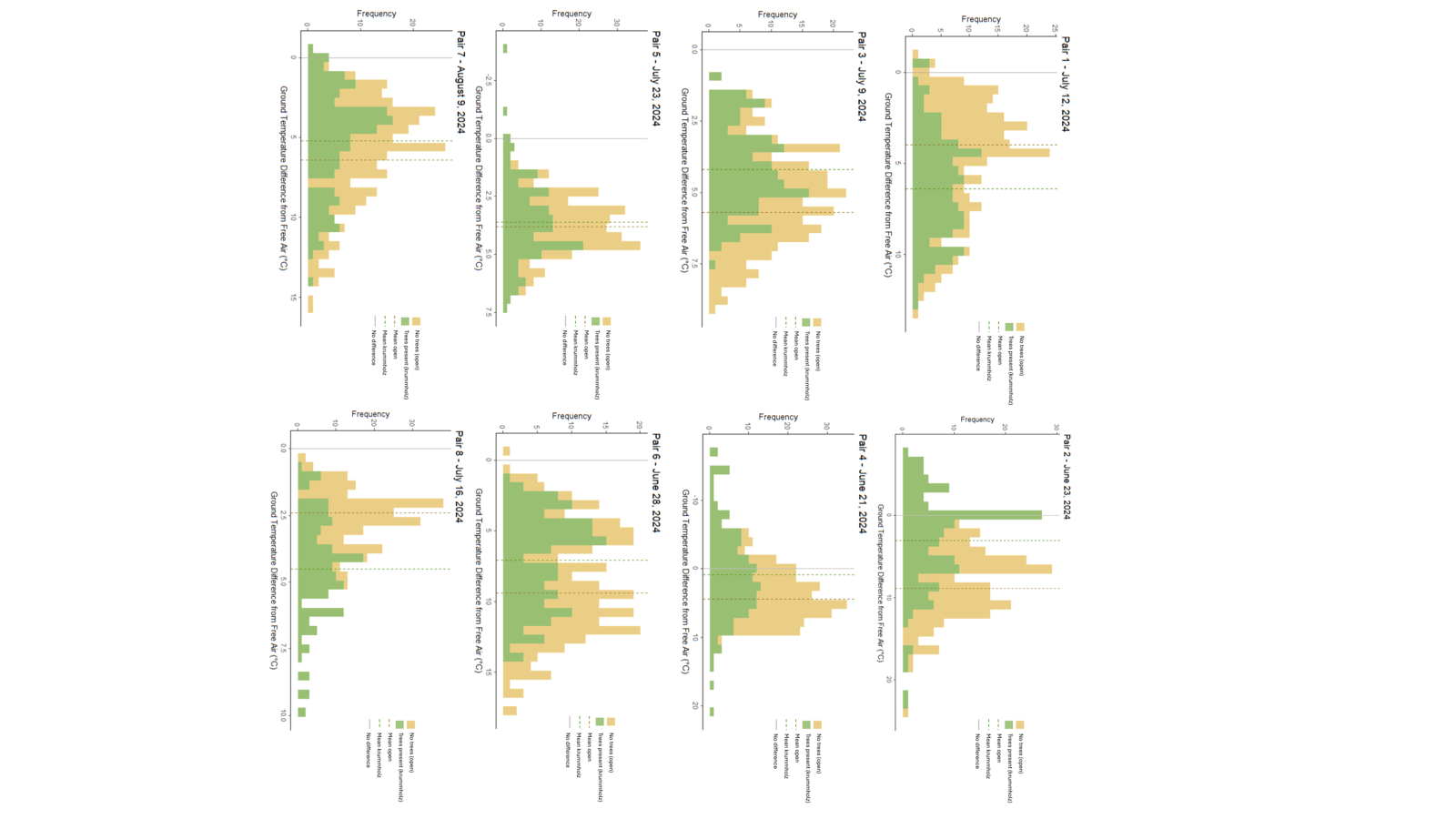
**

**Figure S4.1.** Histogram illustrating the difference in temperatures between surface and free-air for each pair, split according to the presence of krummholz (n = 1) and open alpine (n = 1). The mean of each histogram (treed or open) is shown as a dashed line. Surface and free-air at equilibrium (0 °C) is shown as a solid gray line; the area left of this represents surface cooling; the area to the right represents surface warming.

**Appendix S5.** Temporal Curves of Horizontal Temperature Variability

**
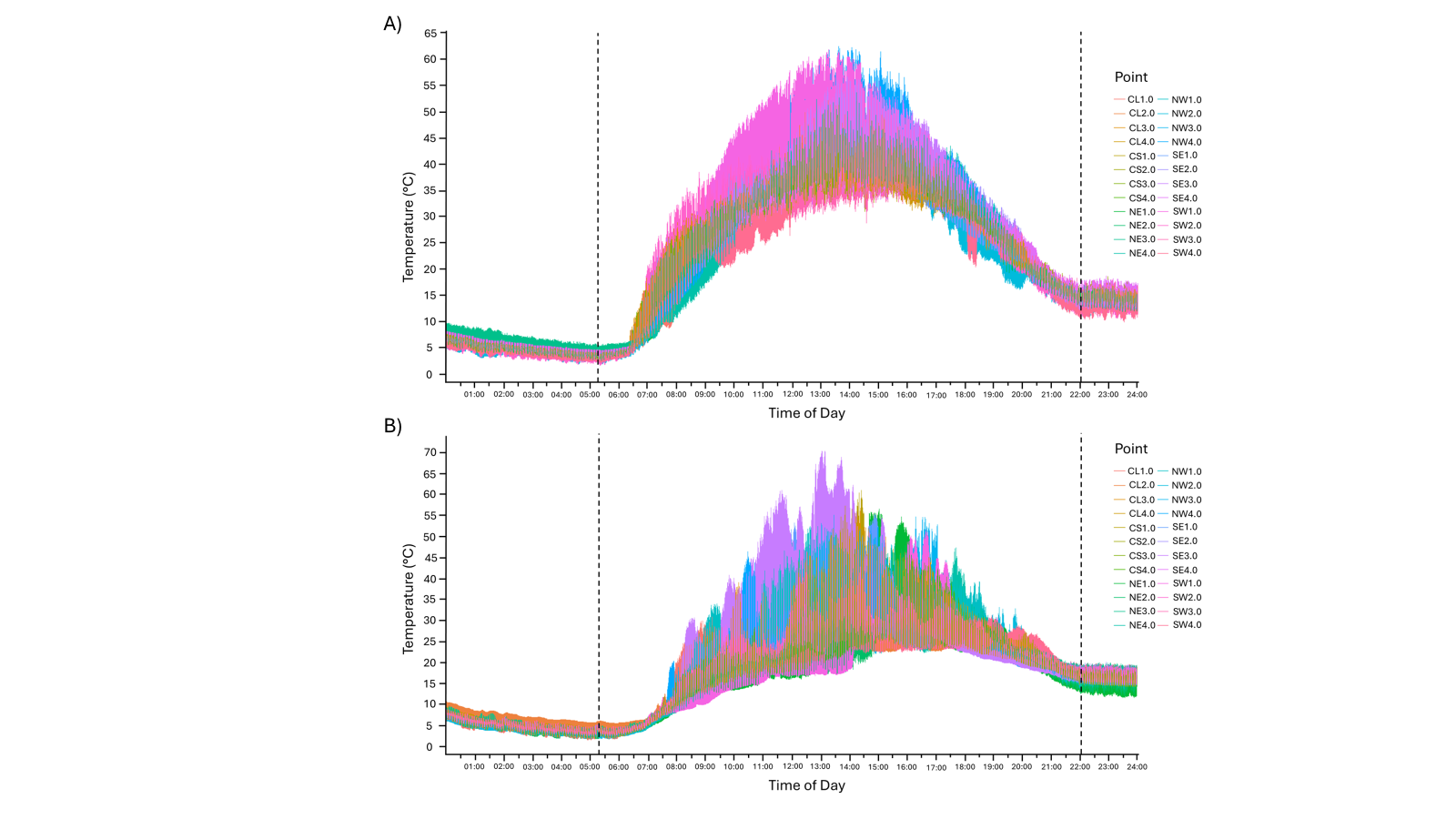
**

**Figure S5.1.** Temperatures for pair 3 plots on July 9, 2024 (the measurement day with the highest mean 24 hr free-air temperature), collected at 24 points within the A) open and B) krummholz plots at Cardinal Divide, Alberta, Canada. The vertical dashed lines represent sunrise and sunset at 05:17 and 22:02, respectively. Data were collected using six four-channel thermocouple (Perfect Prime TC0520) units, allowing for 24 fibreglass-coated K-type wires to be systematically spaced throughout each plot (Figure A1) and used to record diel surface temperatures at 30-second intervals. Plot location within the study site can be found in Figure 1.

| 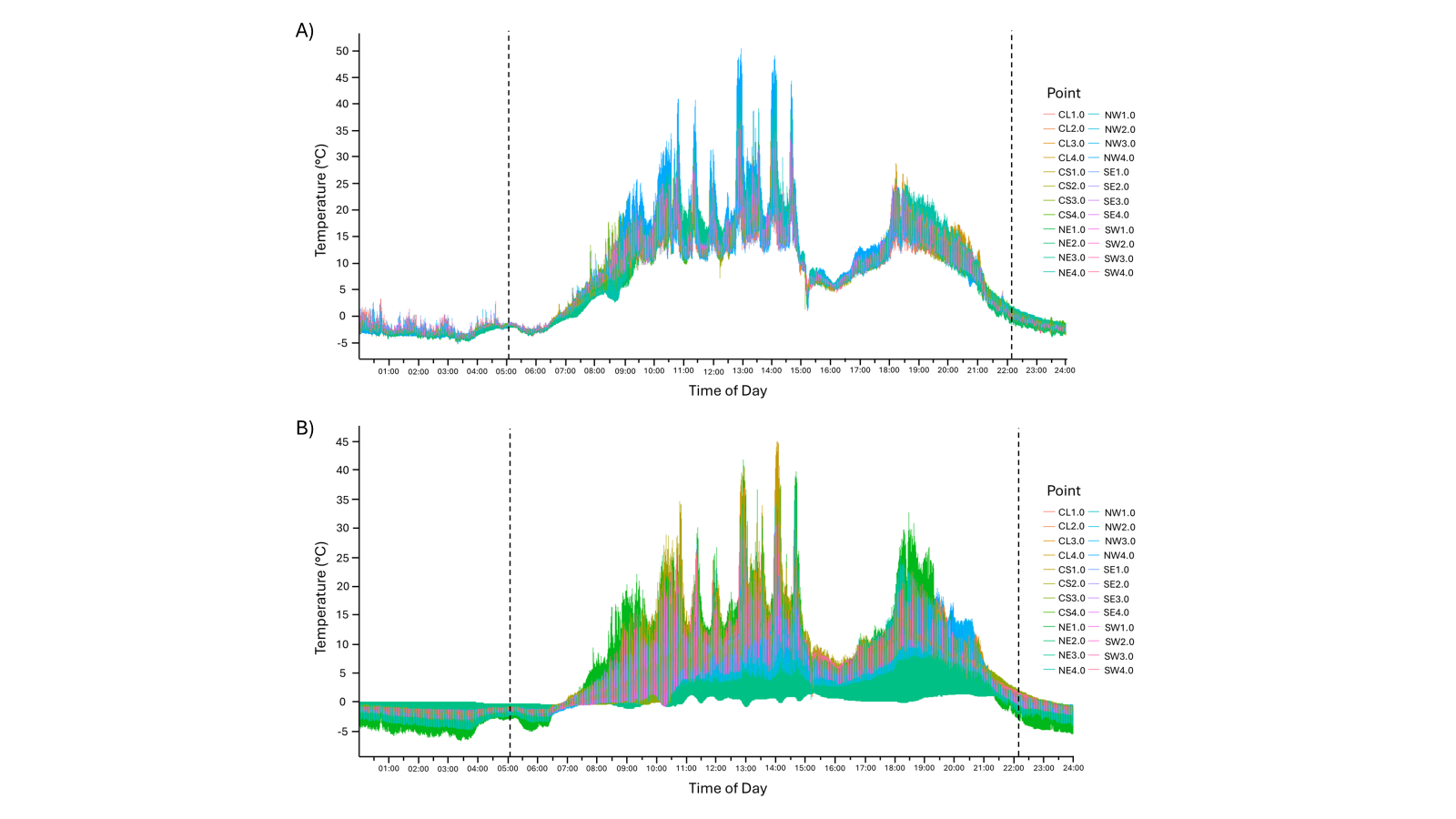 |
| --- |

**Figure S5.2.** Temperatures for pair 4 plots June 20, 2024 (the measurement day with the lowest mean 24 hr free-air temperature), collected at 24 points within the A) open and B) krummholz plots at Cardinal Divide, Alberta, Canada. The vertical dashed lines represent sunrise and sunset at 05:04 and 22:08, respectively. Data were collected using six four-channel thermocouple (Perfect Prime TC0520) units, allowing for 24 fibreglass-coated K-type wires to be systematically spaced throughout each plot (Figure A1) and used to record diel surface temperatures at 30-second intervals. Plot location within the study site can be found in Figure 1.

**Appendix S6.** Surface horizontal microthermal heterogeneity plots.

**
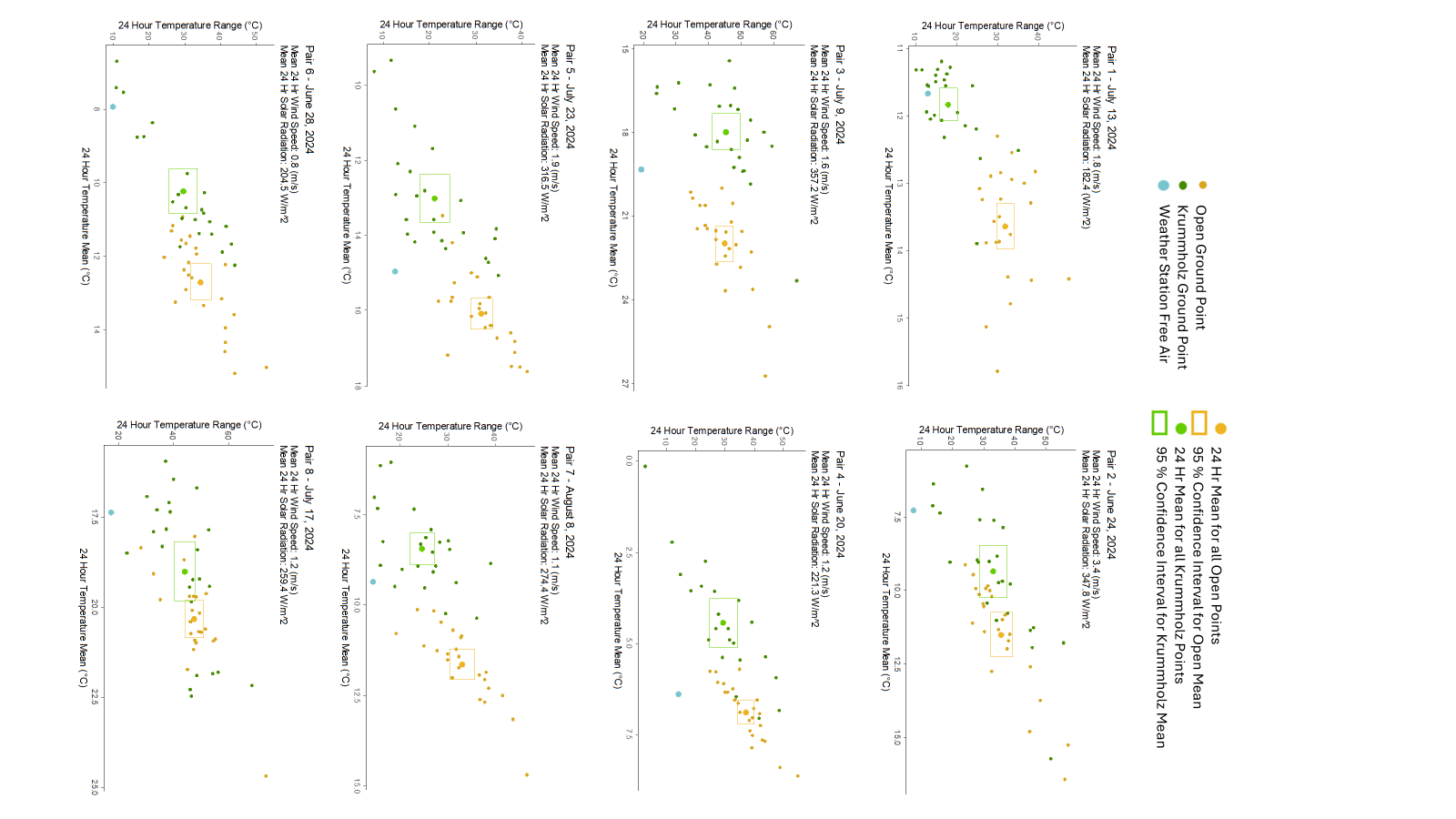
**

**Figure S6.1.** Plots showing the 24-hour range and mean of ground surface temperature for 24 locations per 8x8 m plot. The points are colour-coded such that blue represents plots without trees and green represents plots where krummholz (trees) are present. The mean temperature and range for all open and krummholz points are shown as larger points, with the 95% confidence intervals represented as bounded boxes around the points. As a standard comparison, the mean free-air temperature and range across the same period at the reference weather station are represented by a blue point.
