# Supplementary figures and images for: Alpine Summer Surface Temperature Amplification Is Spatially Heterogeneous and Intensified by Wind and Sun
Source: Ecol Evol. 2025 Nov 23;15(11):e72542. doi: 10.1002/ece3.72542 (PMC12640703; doi:10.1002/ece3.72542)

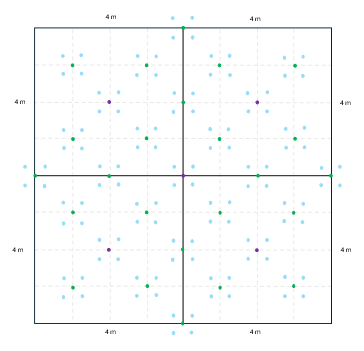

Supplement: Supplementary file 1 — Appendices S1–S6: ece372542‐sup‐0001‐AppendixS1‐S6.zip. [file ECE3-15-e72542-s001.zip › Figure S1.1.png]

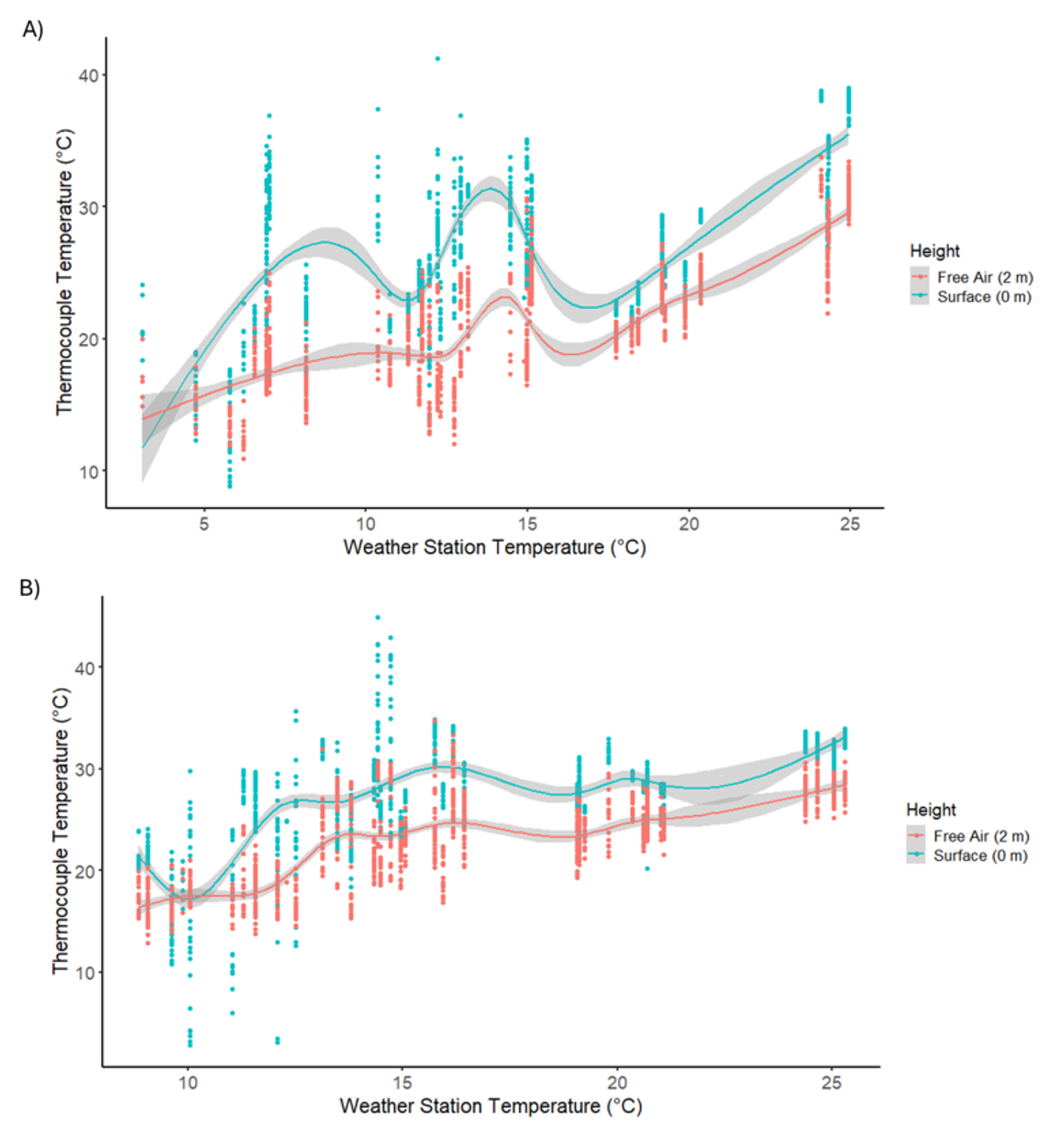

Supplement: Supplementary file 1 — Appendices S1–S6: ece372542‐sup‐0001‐AppendixS1‐S6.zip. [file ECE3-15-e72542-s001.zip › Figure S2.1.png]

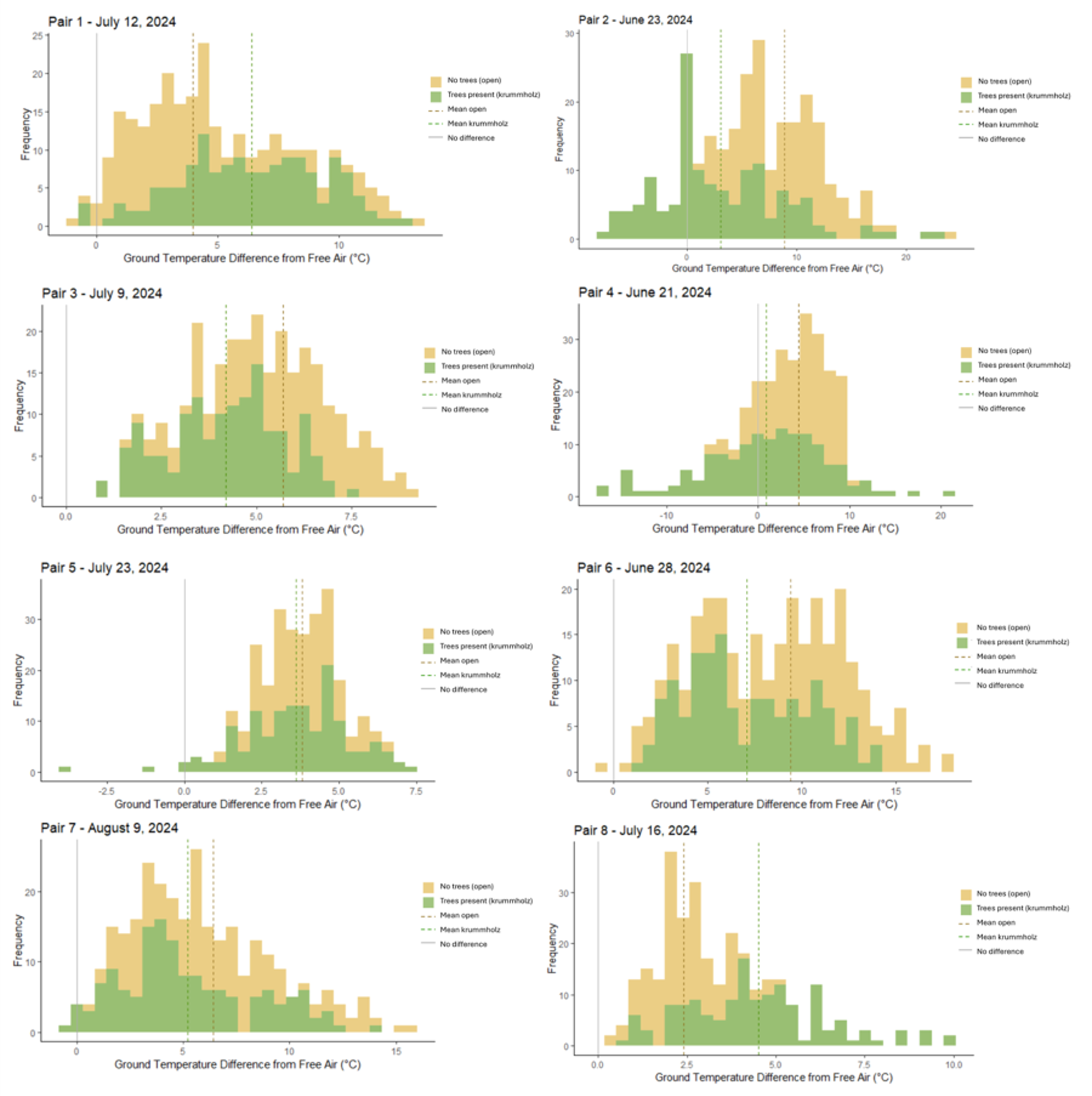

Supplement: Supplementary file 1 — Appendices S1–S6: ece372542‐sup‐0001‐AppendixS1‐S6.zip. [file ECE3-15-e72542-s001.zip › Figure S4.1.png]

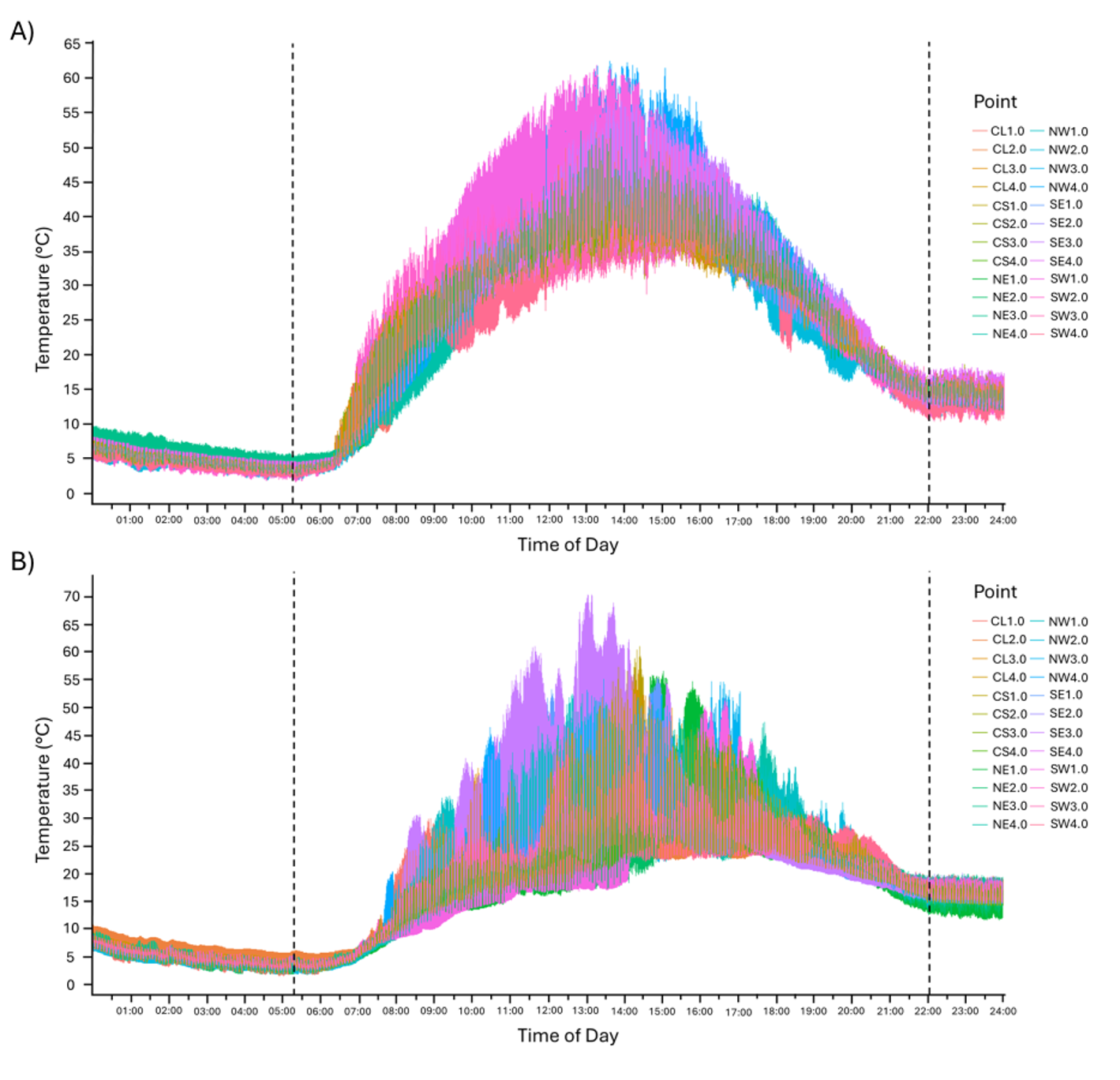

Supplement: Supplementary file 1 — Appendices S1–S6: ece372542‐sup‐0001‐AppendixS1‐S6.zip. [file ECE3-15-e72542-s001.zip › FIgure S5.1.png]

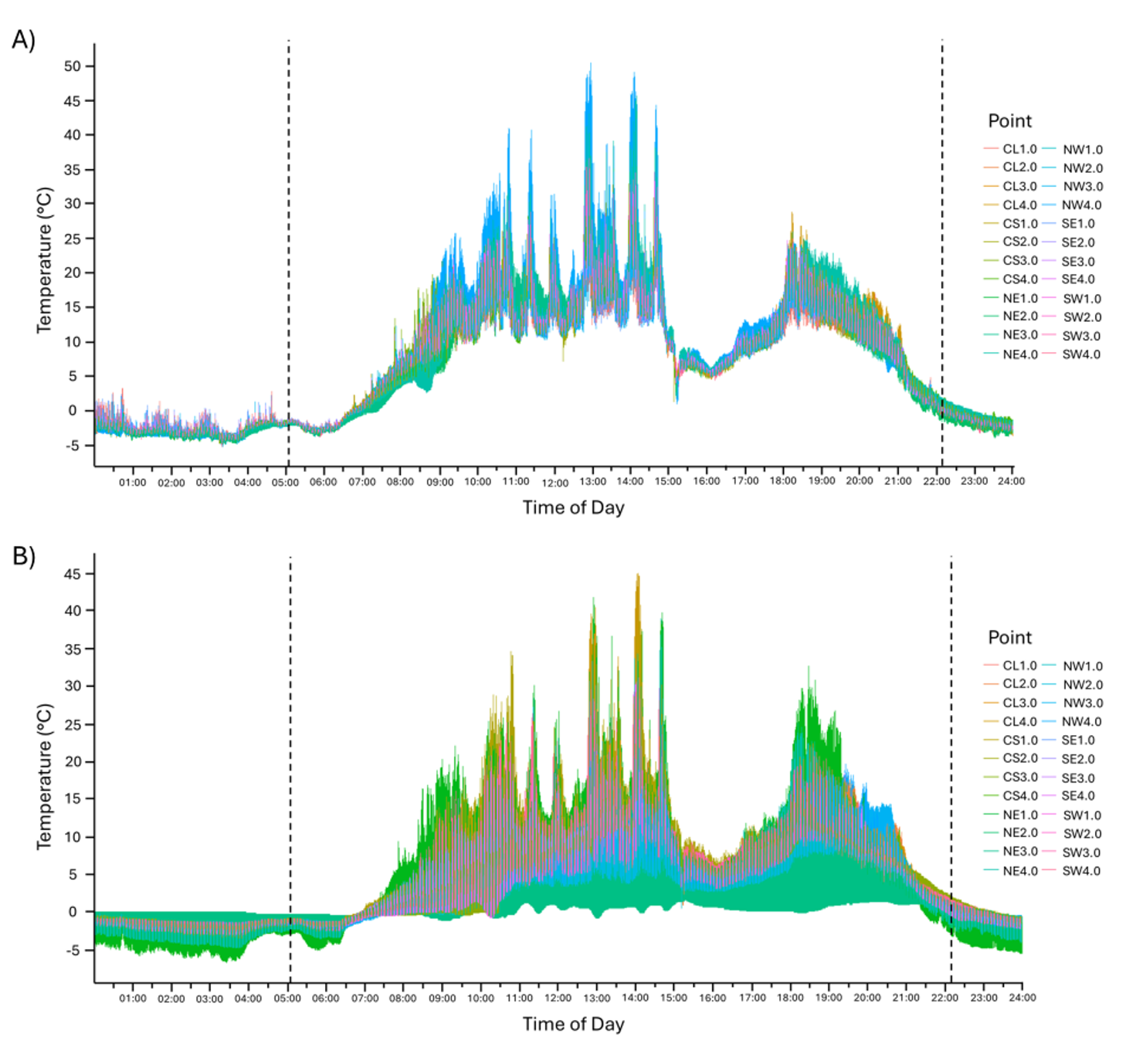

Supplement: Supplementary file 1 — Appendices S1–S6: ece372542‐sup‐0001‐AppendixS1‐S6.zip. [file ECE3-15-e72542-s001.zip › Figure S5.2.png]

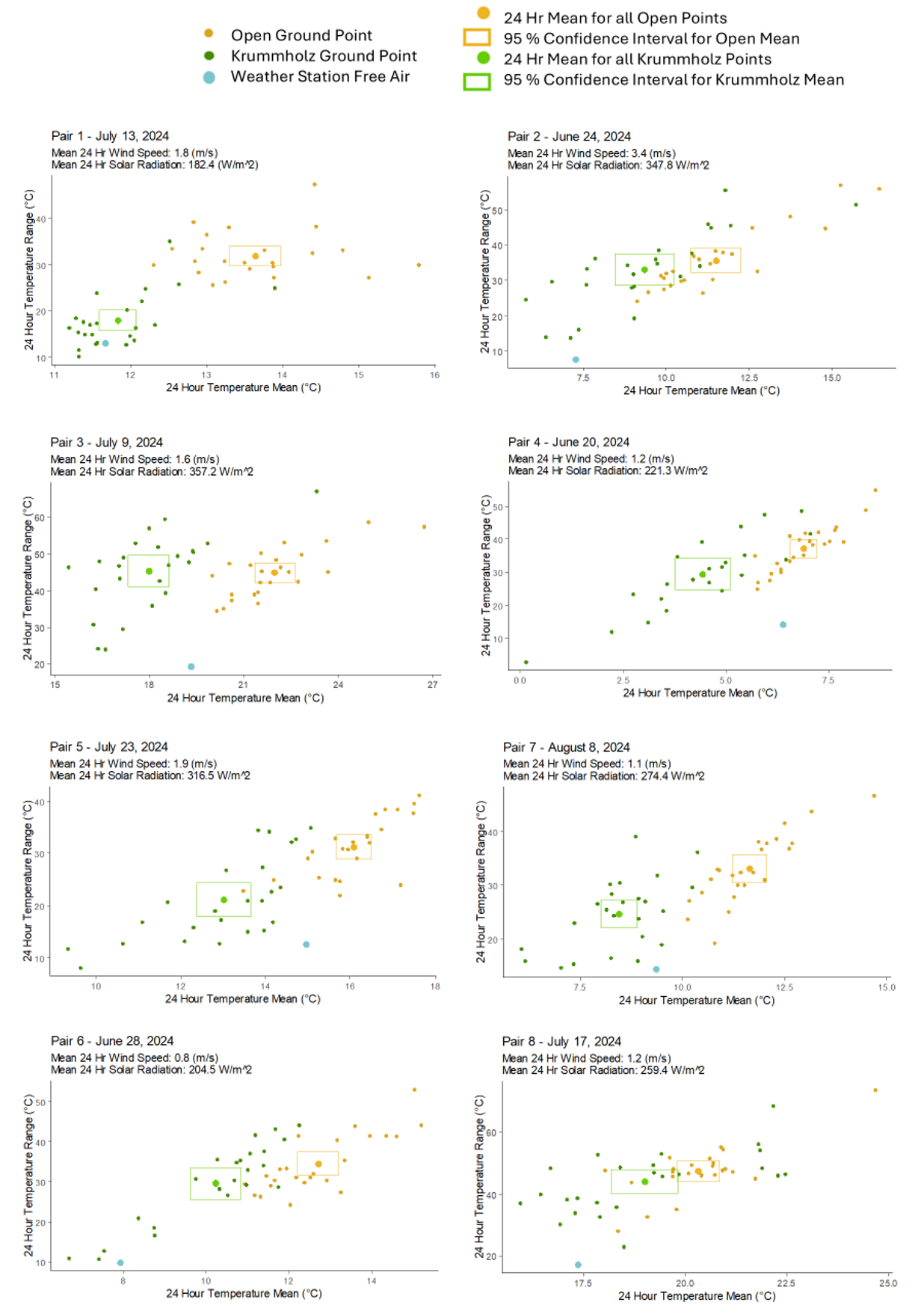

Supplement: Supplementary file 1 — Appendices S1–S6: ece372542‐sup‐0001‐AppendixS1‐S6.zip. [file ECE3-15-e72542-s001.zip › Figure S6.1.png]
